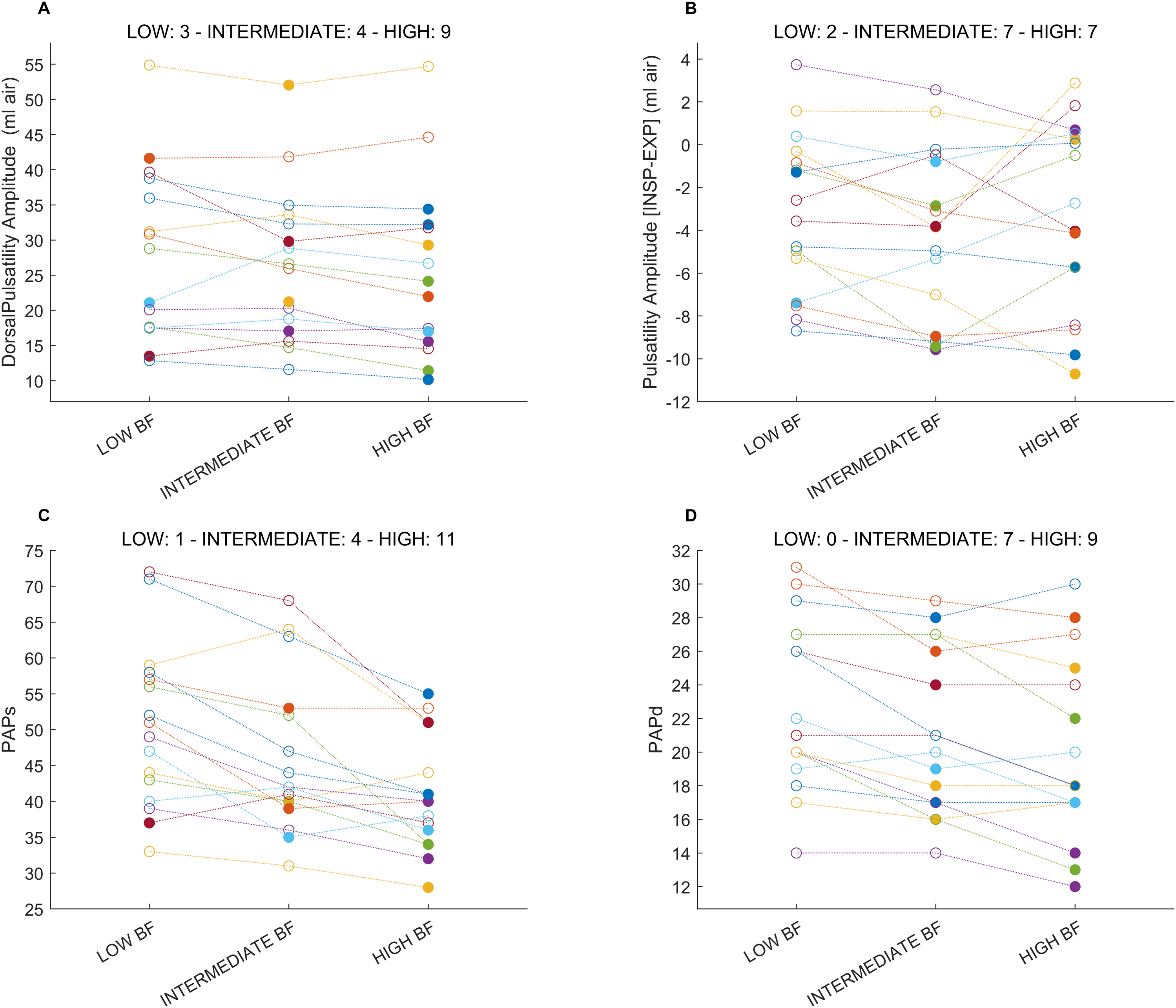# Pulmonary pulsatility quantified by electrical impedance tomography in severe acute respiratory distress syndrome patients undergoing extracorporeal membrane oxygenation support

**DOI:** 10.1016/j.aicoj.2026.100058

**Published:** 2026-03-28

**Authors:** Marco Leali, Elena Spinelli, Marco Giani, Bertrand Pavlovsky, Michela Di Pierro, Stefania Crotti, Alfredo Lissoni, Giuseppe Foti, Giacomo Grasselli, Tommaso Mauri, Douglas Slobod

**Affiliations:** a Department of Pathophysiology and Transplantation, University of Milan, Milan, Italy; b Department of Anesthesia, Critical Care and Emergency, Institute for Scientific Research and Care Foundation Ca’ Granda, Maggiore Policlinico Hospital, Milan, Italy; c Department of Medicine and Surgery, University of Milano-Bicocca, Monza, Italy; d Department of Emergency and Intensive Care, Fondazione Istituto di Ricovero e Cura a Carattere Scientifico San Gerardo dei Tintori, Monza, Italy; e Medical and Toxicologic Intensive Care Unit, Lariboisière Hospital, Assistance Publique Hôpitaux de Paris, Paris, France; f Department of Critical Care Medicine, McGill University, Montreal, Quebec, Canada

**Keywords:** Electrical impedance tomography, Pulsatility, Pulmonary artery pressure, West zones, Extracorporeal membrane oxygenation

## Abstract

**Background:**

The cardiac-related pulsatility signal from electrical impedance tomography (EIT) correlates with stroke volume in mechanically ventilated patients with acute respiratory distress syndrome (ARDS). However, in swine models, regional pulsatility amplitude was also shown to increase with downstream flow obstruction. We aimed to investigate the relationship between regional pulsatility and pulmonary hemodynamics in a cohort of severe ARDS patients on veno-venous extracorporeal membrane oxygenation (ECMO).

**Methods:**

We reanalysed data obtained from 20 ARDS patients receiving ECMO support. EIT was recorded 30 min after adjusting ECMO blood flow to target three ranges of mixed venous oxygen saturation (SvO_2_) (70–75 %; 75–80 %; >80%), applied in random order. Ventilation was protective with PEEP 15[12–16] cmH_2_O, Vt 4 ± 1 ml/kg PBW. Quality of EIT tracings allowed for separate modelling of pulsatility and ventilation in 16/20 patients. EIT units were calibrated to millilitres of tidal volume (ml*) using synchronised tidal volume measurements, allowing for between-patient comparisons. Mixed-effects modelling was employed to account for repeated measurements.

**Results:**

Across blood flow steps, pulsatility amplitude was directly related to stroke volume (SV) (β = 0.28 (0.06 – 0.5) ml*/mL, p = 0.014) and systolic pulmonary artery pressure (PAPs) (β = 0.47 (0.14 – 0.81) ml*/mmHg, p = 0.008) and inversely related to mixed venous oxygen tension (PvO_2_) (β = −0.41 (−0.79–−0.03) ml*/mmHg, p = 0.039). Changes in pulsatility had an 80% concordance rate with changes in SV and 83% with PAPs. At each ECMO blood flow step, there was a decrease in ventral lung pulsatility during inspiration (p < 0.01 for all steps). Moderate to strong correlations were observed between dorsal pulsatility and pulmonary artery pressure (ρ = 0.72, p = 0.001 at Low SvO_2_; ρ = 0.59, p = 0.017 at Intermediate SvO_2_; ρ = 0.81, p < 0.001 at High SvO_2_).

**Conclusion:**

n severe ARDS patients on ECMO, pulsatility amplitude reflects stroke volume changes induced by positive intrathoracic pressures and mixed venous saturation targets. However, downstream flow obstruction appears to be the leading determinant in the dorsal lung and may be useful to monitor right heart loading in patients with ARDS.

## Background

The high temporal resolution of electrical impedance tomography (EIT) makes it well-suited for monitoring regional ventilation in patients with acute respiratory distress syndrome (ARDS) [[1], [2], [3]]. In addition, two other types of functional EIT images can be obtained. Injection of a tracer bolus, most commonly hypertonic saline, can provide information about lung perfusion and ventilation-perfusion matching [[4], [5], [6]]. Similarly, the pulsatile signal related to the heartbeat, normally superimposed on the EIT ventilation signal, has been separated [7,8] and proposed as another way to assess regional lung perfusion [9].

During each cardiac beat, impedance increases in the cardiac region and decreases shortly after in the lung regions. It is therefore assumed that the pulsatility signal may be due to blood flowing from the heart through the lungs. Indeed, EIT pulsatility is directly related to stroke volume [[10], [11], [12]].

However, the change in vessel size that gives rise to pulsatility is also related to pulmonary vessel compliance [13]. In addition, a paradoxical increase in pulsatility was observed after injecting microspheres into the pulmonary circulation [5], and higher pulsatility values were also observed in collapsed lung regions of swine ARDS models, whereas regional blood flow, was shown to be reduced in both settings [4]. These considerations suggest that pulsatility results from a complex interaction between inflow into the pulmonary circulation (i.e. right ventricular (RV) stroke volume) and the properties of downstream pulmonary vessels.

In ARDS patients, several mechanisms induce downstream flow obstruction indicating critical closure [14], an increase in vascular resistance, or a decrease in the cross-sectional area of pulmonary vessels. These occur due to West non-Zone 3 conditions [15], microvascular and macrovascular thrombosis [16] and hypoxic pulmonary vasoconstriction [17]. Regional quantification of the hemodynamic impact of these phenomena could provide insights into the determinants of ARDS pathophysiology, which are difficult to assess at the bedside [18].

Downstream flow obstruction increases the load opposing RV ejection and often represents a major factor that limits cardiac output in critically ill patients. Monitoring and management of right ventricular afterload is of significant importance, even when right ventricular contractility is preserved [18,19] but significant knowledge gaps regarding the characterization and monitoring of RV injury remain [20,21]. Although acute cor pulmonale portends increased mortality in ARDS [22,23] bedside tools for monitoring RV afterload, such as pulmonary artery catheters or echocardiography, are invasive and/or require trained personnel, limiting their applicability [24,25].

We performed a secondary analysis of a study on patients with severe ARDS undergoing extracorporeal membrane oxygenation (ECMO), which included EIT monitoring [26]. We expected this population to be particularly prone to downstream flow obstruction due to the above listed mechanisms. In addition, physiologic measurements were taken at 3 distinct, pre-defined mixed venous saturation (SvO_2_) targets obtained by titrating ECMO blood flow, resulting in the hemodynamic changes described previously [26]. This cohort was, therefore, suitable for studying the relationship between pulsatility, oxygenation and its effects on the pulmonary and systemic circulations. We hypothesized that downstream pressure increases due to West non-Zone 3 conditions could be detected by analyzing pulsatility changes during the respiratory cycle, while downstream flow obstruction due to ARDS pathophysiology could be observed between patients and modified by ECMO blood flow.

## Methods

### Protocol

This is a secondary analysis of a prospective physiologic randomized crossover study of twenty patients undergoing veno-venous extracorporeal membrane oxygenation (ECMO) for severe acute respiratory distress syndrome (ARDS). The study was conducted in two Italian university hospitals. Ethical approval and informed consent were obtained according to local regulations. For further information, please refer to the original publication [26]. Briefly, enrolled patients were intubated, deeply sedated, paralyzed, on controlled mechanical ventilation with a pulmonary artery catheter in place. Clinical settings for ventilation and sweep gas flow (PEEP 15 [[12], [13], [14], [15], [16]] cmH_2_O, Vt/PBW 4 ± 1 ml/kg, RR 12 ± 0.5 breaths/min, plateau pressure 27 [[5], [6], [7], [8], [9], [10], [11], [12], [13], [14], [15], [16], [17], [18], [19], [20], [21], [22], [23], [24], [25], [26], [27], [28], [29]] cmH_2_O, FiO_2_ and FdO_2_ 66 ± 16%, sweep gas flow 4.5 (3.8–6.5) l/min) were maintained throughout the study whereas ECMO blood flow was adjusted to target three distinct SvO_2_ ranges, in a randomized order:

• Low SvO_2_: ECMO blood flow was set to obtain a SvO_2_ of 70–75%.
• Intermediate SvO_2_: ECMO blood flow was set to obtain a SvO_2_ of 75–80%.
• High SvO_2_: ECMO blood flow was set to obtain a SvO_2_ >80%.

As shown in the original publication [26] and summarised in the Results section, these SvO_2_ targets resulted in significant hemodynamic changes. The relationship between these hemodynamic changes and EIT pulsatility is the focus of this secondary analysis.

The steps lasted 30 min, after which ventilatory data, hemodynamics, blood gas analyses and electrical impedance tomography were collected.

Electrical impedance tomography traces were recorded for an average of 6 min at the end of each step. A 16-electrode belt was positioned on the 5–6th intercostal space and connected to an EIT monitor (PulmoVista 500, Draeger, Lübeck). EIT images were sampled at 50 Hz and reconstructed via Draeger’s proprietary algorithm [1].

### Pulsatility separation

The relationship between the cardiac-related pulsatile impedance signal and the phase of the respiratory cycle is a well-known phenomenon [27,28]. This was particularly evident in our data, likely due to enhanced heart-lung interactions in severe ARDS patients. The phase-lock induced by this phenomenon and the high-frequency components in the ventilatory signal, resulting from the ventilation in pressure control mode of lungs with low compliance, prevented us from employing some of the more commonly employed algorithms for the separation of the pulsatile and respiratory signals in electrical impedance tomography traces [8,9]. In addition, as our study aimed to explore heart-lung interactions, we sought to study respiratory variations in pulsatility with increased accuracy.

Therefore, we modified a previously published algorithm, known as the modified Schuessler-Bates Method (mSBM) [28,29], initially conceived to separate the pulsatility signal from ventilation in impedance pneumography. This was shown to outperform high-pass filtering and was specifically designed to account for respirophasic changes in pulsatility.

**Fig. 1:**
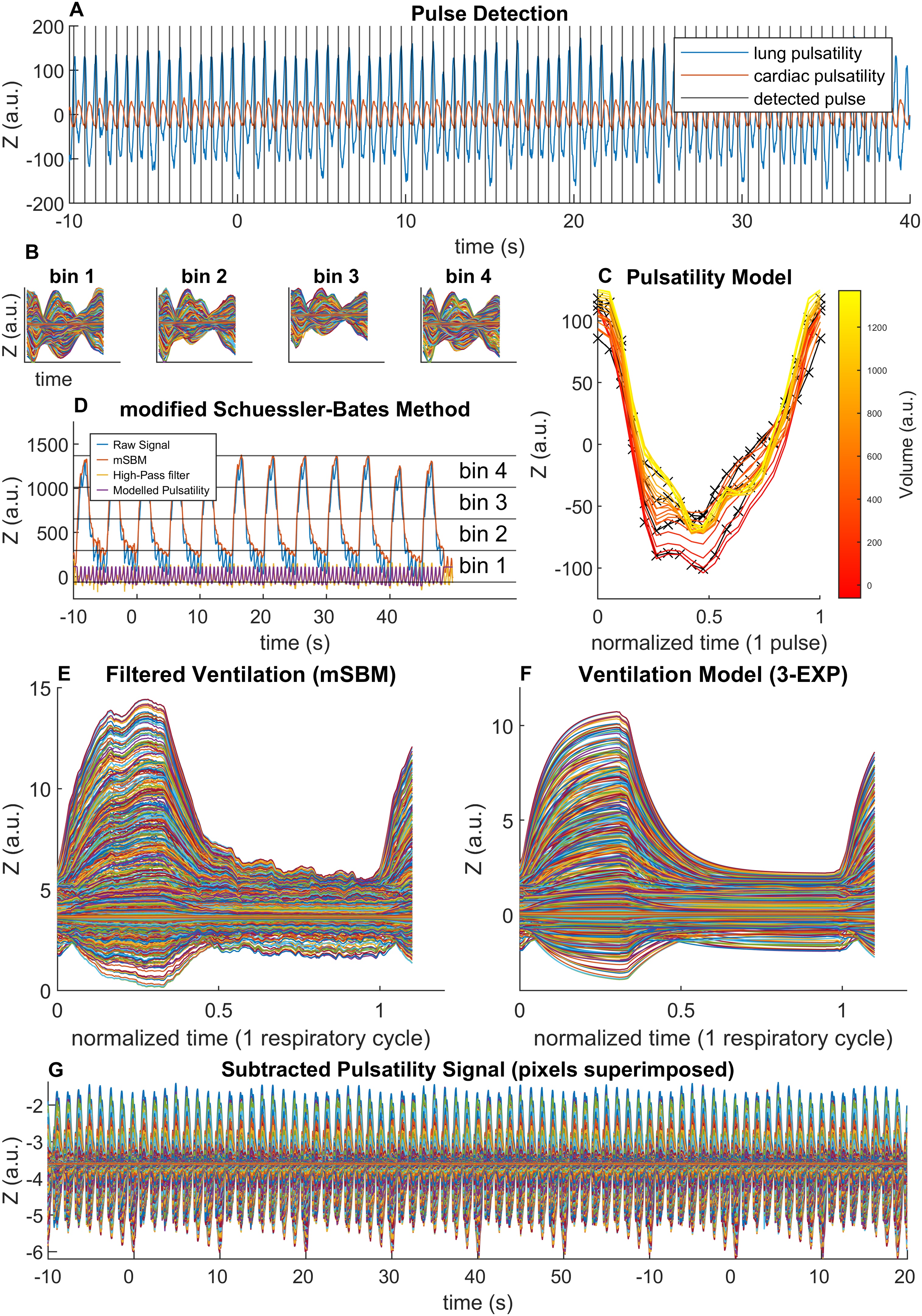
Model-based algorithm employed to separate the pulsatility and the ventilation signals obtained by electrical impedance tomography (EIT). The algorithm is shown for one representative patient. **A** – The cardiac and lung regions of interest (ROI) were separated based on their phase. In 16/20 patients the cardiac pulsatility signal was clearly discernible and its local minima were used as a trigger for ensemble averaging. **B,C,D** – According to Seppä et al. [28] and using the cardiac signal as a reference, the EIT pulses were averaged in four ensembles (bin 1-4), corresponding to four increasing lung volumes. In panel **B** the four averaged pulses are shown. All pixels have been here superimposed. From the ensemble averages, a lung volume-dependent piecewise linear model of the EIT pulse is developed for each pixel. Panel **C** portrays the average of all pixel models across the spectrum of observed lung volumes (colour scale): black lines are the observed data, red/yellow lines represent the interpolated data. In panel **D** pixels have been averaged over time to show the raw signal (*blue*) and the filtered signal (*orange*), resulting from the modified Schuessler-Bates Method (mSBM) above described. The modelled pulsatility signal, averaged across all pixels, is shown at the bottom (*purple*) and compared to the signal that would be obtained by conventional high-pass filtering (*yellow*). **E** – Respiratory cycles, after being filtered by the mSBM algorithm, are then averaged to obtain a representative act. Signals from all pixels have been here superimposed. **F** – A model consisting of three exponential terms (3-EXP) is fit to each pixel. Please note that part of the pulsatility signal is still retained into the filtered data. **G** – The ventilation model is subtracted from raw data and the remaining signal is taken as pulsatility. Signals from all pixels have been here superimposed. Impedance (Z) is expressed in arbitrary units (a.u.).

In place of electrocardiography gating, we used the pulsatile signal in the cardiac region of interest (ROI) (Fig. 1A). First, we high-pass filtered the impedance signal with a zero-phase 2nd order Butterworth filter, using 60% of the patient’s heart rate, as obtained from the patient's monitor, as the cut-off frequency. This filtering was only used for the purpose of pulse detection, while the mSBM method was applied to the raw signal. We then separated the cardiac and lung ROIs according to their phase relative to the global impedance waveform, as detailed by Frerichs et al., 2009 [9]. Only pixels with a signal range (i.e., a rough estimate of pulsatility) >20% of the maximum value within the image were considered for pulse detection. The summation of the pixels in the cardiac ROI was then used as a reference: the local minima of the summed signal were used as triggers for ensemble averaging in the mSBM algorithm. The detection of local minima was a two-step procedure, which made use of matched filter event detection, a technique proposed to increase accuracy in impedance cardiography [27]. First, the actual cardiac period was estimated by cross-correlation of the high-pass filtered trace. Then a local minima search (MatLab's built-in algorithm *findpeaks*) was run, using the estimated cardiac period ±100 ms as a constraint. Finally, the detected minima were used to create a one cardiac period template, which was used for matched filter event detection, as detailed in Nagel et al., 1989 [27].

Strict quality criteria were applied to pulse detection. From heart rate variability studies [[30], [31], [32]] the percentage of consecutive normal-to-normal (NN) beats differing by more than 100 ms approaches zero and the standard deviation of normal-to-normal (SDNN) beat durations over a few minutes of recording is expected to be lower than 100 ms. Beats that failed to fulfil either of these two criteria were rejected and the corresponding pulse was linearly interpolated. Patients in whom more than 30% of pulses had to be interpolated, due to low-quality or noisy pulsatility signals, were excluded from this analysis.

Once reliable pulse detections were obtained, the modified Schuessler-Bates Method (mSBM) was applied to the raw signal [28]. Ensemble averaging using a pulse signal as the trigger is performed over a discrete number (four) of volume ranges across the respiratory cycle (Fig. 1B,D). Then, the four ensembles are used to derive a piecewise, 20-sample linear model of how the pulsatile signal changes throughout the respiratory cycle (Fig. 1C) for any given volume. Finally, the model is applied for each detected pulse and subtracted from the raw data (Fig. 1D). This process was performed for each pixel of our EIT images, thus obtaining a filtered regional ventilation signal, where cardiac pulsatility had been suppressed, but where high-frequency ventilation components were still discernible (Fig. 1E).

Finally, to further recover the remaining part of the pulsatility signal which could not be considered by the mSBM pulse model, the ventilation signal was modelled based on known physiology. As a two-compartment model is required to describe a healthy lung [33], and a third exponential term can be used to account for lung recruitment [34], we averaged all the respiratory cycles and, for each pixel, we fit data to a theoretical model consisting of three exponential terms and an intercept (Fig. 1F). A non-iterative linearized method for exponential fitting was used [35]. Then, the obtained parameters were used as a starting point for an iterative nonlinear least squares solver (trust-region-reflective algorithm), that fit the same three-exponential model (3-EXP) for each breath, in each pixel. To account for the transition between expiration and inspiration, a smoothing function (smoothing spline) was fit to data for 100 ms around the transition. The pulsatility signal was then obtained by subtracting the modelled ventilation signal from the raw data (Fig. 1G).

### EIT metrics

The resulting pulsatility signal was then processed according to the same pulse reference used for the separation algorithm to obtain a cardiac and a pulmonary region of interest (ROI) [36,37]. However, lacking proper ECG gating, we refrained from employing the discrete Fourier transform and simply calculated the pulse amplitude and its phase as the slope during the first half of the cardiac cycle.

The ventilation signal ranges from end-inspiratory to end-expiratory lung volumes. A midpoint between the two was calculated, so that each EIT frame could be categorized as occurring during end-expiration or end-inspiration. An EIT pulse was categorized as obtained at end-inspiration (INSP) if at least 75% of its frames corresponded to ventilation frames in the upper half of the ventilation signal, while pulses with at least 75% of their frames corresponding to ventilation frames in the lower half, were categorized as obtained at end-expiration (EXP). Consequently, some pulses occurring at intermediate lung volumes were excluded from the analysis. The percent variation of pulsatility over the respiratory cycle was calculated as ${100\text{*}(\text{Δ}Z}_{EXP}-{\text{Δ}Z}_{INSP)}/\text{Δ}Z$, where ${\text{Δ}Z}_{EXP}$ and ${\text{Δ}Z}_{INSP}$ stand for pulse amplitude during the corresponding respiratory phases and ΔZ stands for average pulse amplitude.

To allow for between-patient comparisons, EIT arbitrary units were calibrated by comparing the tidal impedance variation to the tidal volume measured with the ventilator.

### Statistics

Continuous data are shown as mean ± standard deviation, or as median (interquartile range), as appropriate. Qualitative data are shown as count (percentage). Normality was tested by the Lilliefors test. Comparisons between end-inspiratory and end-expiratory variables were carried out by paired t-tests, or Wilcoxon tests, as appropriate. Comparisons between the three SvO_2_ steps were carried out by one-way repeated measures analysis of variance (rmANOVA), or by Friedman tests, as appropriate. Correlations were expressed as Pearson’s r or Spearman’s rho, depending on the distributions of the correlated variables. Mixed-effects models with the individual patients as random intercepts and physiological variables as fixed effects were fitted to data. The percentage of total variability explained by fixed effects was calculated by computing marginal R^2^ values, as previously proposed [38]. F-tests using Satterthwaite's approximation [39] were used to assess the significance of the fixed-effect coefficients. Four quadrant plots were drawn and the concordance rate (CR) was calculated as previously described [40]. A discordance rate (DR) was calculated in an analogous way for inversely related quantities. A 10% exclusion zone for relative changes was adopted [41].

### Software

Image processing and statistical analyses were performed with MatLab R2024b (MathWorks Inc., Natick, Massachusetts). Mixed effect modelling was performed with R version 4.4.3 [42]. Packages *lme4* [43] and *lmerTest* [44] were used.

## Results

### Patients

Traces from four patients did not meet quality criteria for pulse detection and were excluded. Sixteen of twenty (80%) patients could be analysed, corresponding to 48 EIT recordings. In 31/48 (65%) traces, all detected beats fulfilled the quality criteria. Overall, 0% (0%–4%) beats required interpolation and the percentage of interpolated beats exceeded 10% in 3 patients, reaching a maximum of 29% in one trace.

Baseline characteristics of the analysed cohort are shown in Table 1. The ARDS etiology was infectious in all patients: in 11 (69%) it was primarily attributed to Sars-Cov-2, and in 5 (31%) to bacterial pneumonia. Before ECMO initiation, the PaO_2_/FiO_2_ was 80 ± 16 mmHg and PaCO_2_ was 61 ± 10 mmHg.

**Table 1:** Baseline patient characteristics and physiological trends. Baseline patient characteristics and ECMO, ventilatory and hemodynamic variables obtained during the experimental protocol in the n = 16 patients whose electrical impedance tomography (EIT) traces were reanalysed.

n = 16	Baseline			
Age (years)	52 ± 10			
Gender (M/F)	6/10			
ARDS etiology	Bacterial Pneumonia 5 (31%) COVID-19 11 (69%)			
SOFA at enrolment	5 (4–8.5)			
Days of intubation before ECMO	3.5 (2–5)			
ECMO configuration	Femoro-femoral 5 (31%) Femoro-jugular 11 (69%)			
Gauge of femoral drainage (Fr)	25 ± 1			
P/F before ECMO (mmHg)	80 ± 16			
PaO_2_ before ECMO (mmHg)	65 ± 10			
PaCO_2_ before ECMO (mmHg)	61 ± 10			
pHa before ECMO	7.32 ± 0.07			

Step	Low SvO_2_	Intermediate SvO_2_	High SvO_2_	p-value
ECMO BF (l/min)	1.51 (1.17–1.90)	2.50 (2.14–2.98)	3.48 (3.25–3.81)	<0.001
ECMO BF/CO	0.17 (0.11−0.23)	0.31 (0.23−0.36)	0.42 (0.37−0.48)	<0.001
ECMO drainage pressure (mmHg)	11.8 ± 20.4	0.4 ± 29.0	−23.7 ± 25.6	<0.001
PEEP (cmH_2_O)	15 (14–16)	15 (14–16)	15 (14–16)	1
Vt/PBW (ml/kg)	3.1 ± 1.4	3.2 ± 1.4	3.2 ± 1.4	0.155
CO (l/min)	9.7 (8.2–11.3)	8.5 (8.1–10.1)	8.3 (7.6–9.1)	0.003
SV (ml)	97.9 (93.7–106.6)	88.8 (83.1–98.3)	87.8 (80.3–94.3)	0.002
HR (bpm)	99.5 (82–109)	100.5 (83–107)	100.5 (78–109.5)	0.339
MAP (mmHg)	82.5 (79.5–97)	84.5 (80.5–90)	79 (73.5–86)	0.156
PAPd (mmHg)	22.9 ± 5.1	21.3 ± 4.9	20.0 ± 5.5	<0.001
PAPm (mmHg)	32 (28.5–39.5)	30 (26.5–36)	28 (26.5–32.5)	<0.001
PAPs (mmHg)	50.5 ± 11.3	46.1 ± 11.0	40.9 ± 8.0	<0.001
CVP (mmHg)	11.4 ± 4.3	10.4 ± 4.6	10.2 ± 4.2	0.049
PAOP (mmHg)	12.5 (9.5–17)	12 (10–15.5)	11 (10–14.5)	0.148

ARDS – acute respiratory distress syndrome; BF – blood flow; CO – cardiac output; CVP – central venous pressure; ECMO – extracorporeal membrane oxygenation; HR – heart rate; MAP – mean arterial pressure; P/F – ratio between arterial oxygen tension and inspired fraction of oxygen; PaCO_2_ – arterial carbon dioxide tension; PaO_2_ – arterial oxygen tension; PAOP – pulmonary artery occlusion pressure; PAPd – diastolic pulmonary artery pressure; PAPm – mean pulmonary artery pressure; PAPs – systolic pulmonary artery pressure; PEEP – positive end-expiratory pressure; PBW – predicted body weight; pHa – negative logarithmic hydrogen ion concentration; SOFA – sequential organ failure assessment; SV – stroke volume; Vt – tidal volume.

The physiological changes that occurred during modification of ECMO blood flow have been previously published [26] and are briefly summarized in Table 1. PEEP was 15 [[14], [15], [16]] cmH_2_O and driving pressure and respiratory rate were set to 12 cmH_2_O and 10–12 breaths per minute, respectively. Ventilation was ultra-protective, with mean Vt/PBW 3.13–3.19 ml/kg across the study steps. With increasing ECMO blood flow (BF), a decrease in cardiac output (from 9.65 (8.15–11.28) to 8.33 (7.63–9.10) l/min, p < 0.001) was observed that was mainly due to a reduction in stroke volume from 97.87 (93.66–106.61) to 87.77 (80.30–94.34) ml (p = 0.002). There was a significant decrease in all pulmonary artery pressures (p < 0.001 for PAPs, PAPm and PAPd).

### Pulsatility amplitude and stroke volume

After calibrating each patient’s EIT tracings to millilitres of tidal volume (ml*), correlation between stroke volume and pulsatility amplitude was weak and nonsignificant (ρ = 0.29, p = 0.278 at Low SvO_2_; ρ = 0.35, p = 0.184 at intermediate SvO_2_; ρ = 0.34, p = 0.196 at high SvO_2_).

### Pulsatility variation over the respiratory cycle

**Fig. 2:**
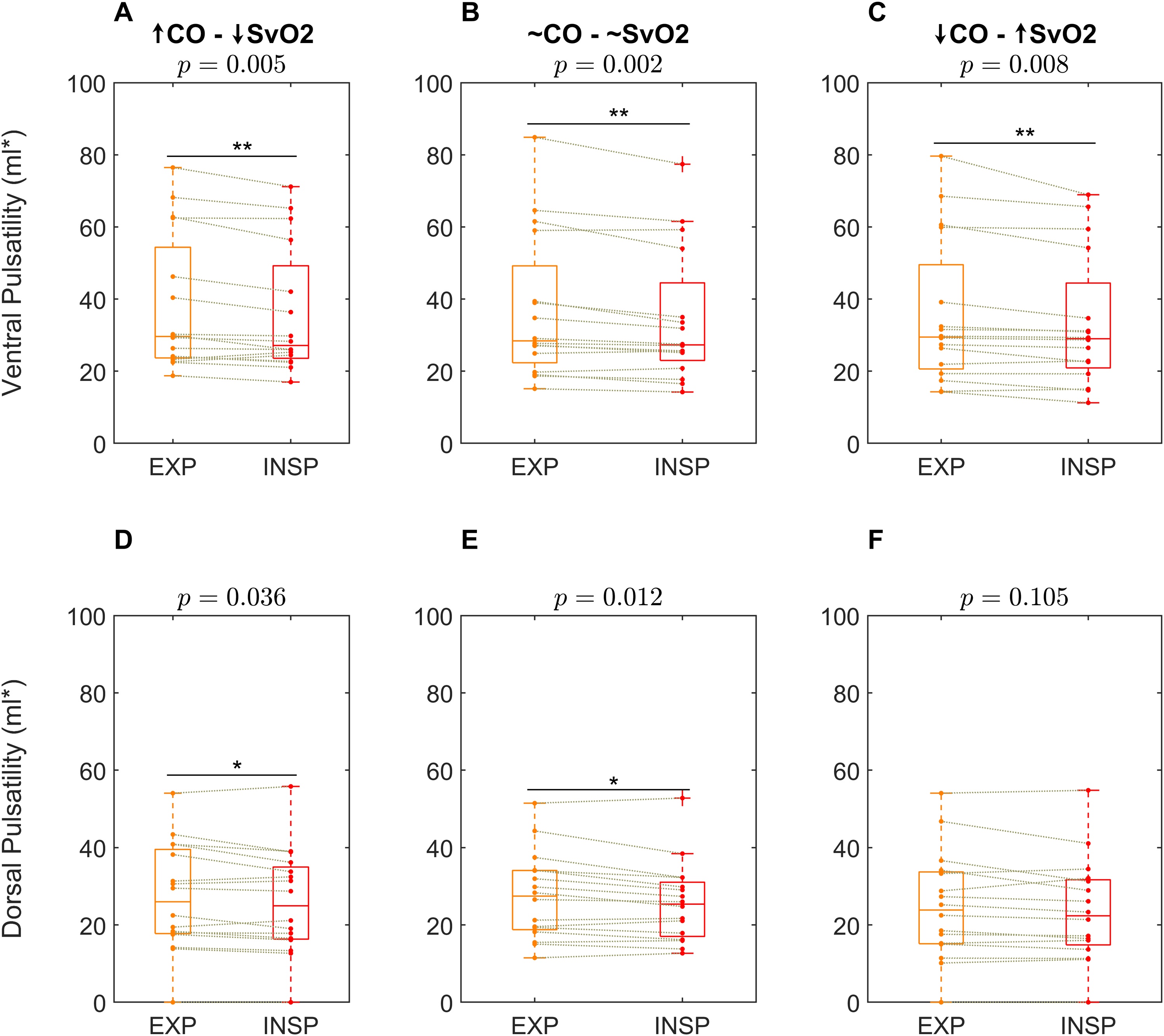
Respiratory variations of pulsatility. Data were collected for each patient during three extracorporeal membrane oxygenation (ECMO) blood flow settings, which were shown to affect both mixed venous oxygen saturation (SvO_2_) and cardiac output (CO) and thus venous return [26]. The *left column* corresponds to the lowest ECMO blood flow, targeting SvO_2_ values in the 70-75% range and resulting in a cardiac output of 9.65 (8.15 - 11.28) l/min. The *middle column* refers to a 75-80% SvO_2_ target and a cardiac output of 8.50 (8.08 - 10.10) l/min, and the *right column* to a SvO_2_ >80% target with a cardiac output of 8.33 (7.63 - 9.10) l/min. **A-C** - in the ventral lung, a decrease in pulsatility amplitude at inspiratory lung volumes (INSP), as compared to expiratory lung volumes (EXP) was consistently observed at the three steps. **D-F** – in the dorsal lung, a significant difference was observed only during the low and intermediate SvO_2_ steps. * p < 0.05; ** p < 0.01.

At all ECMO blood flow steps, pulsatility amplitude in the ventral lung decreased at end-inspiration (p < 0.01 at all steps, Fig. 2A–C). The same trend was present but less pronounced in the dorsal lung at the low (p = 0.036) and intermediate SvO_2_ (p = 0.012) steps, not reaching statistical significance at the high SvO_2_ step (p = 0.105) (Fig. 2D–F).

**Fig. 3:**
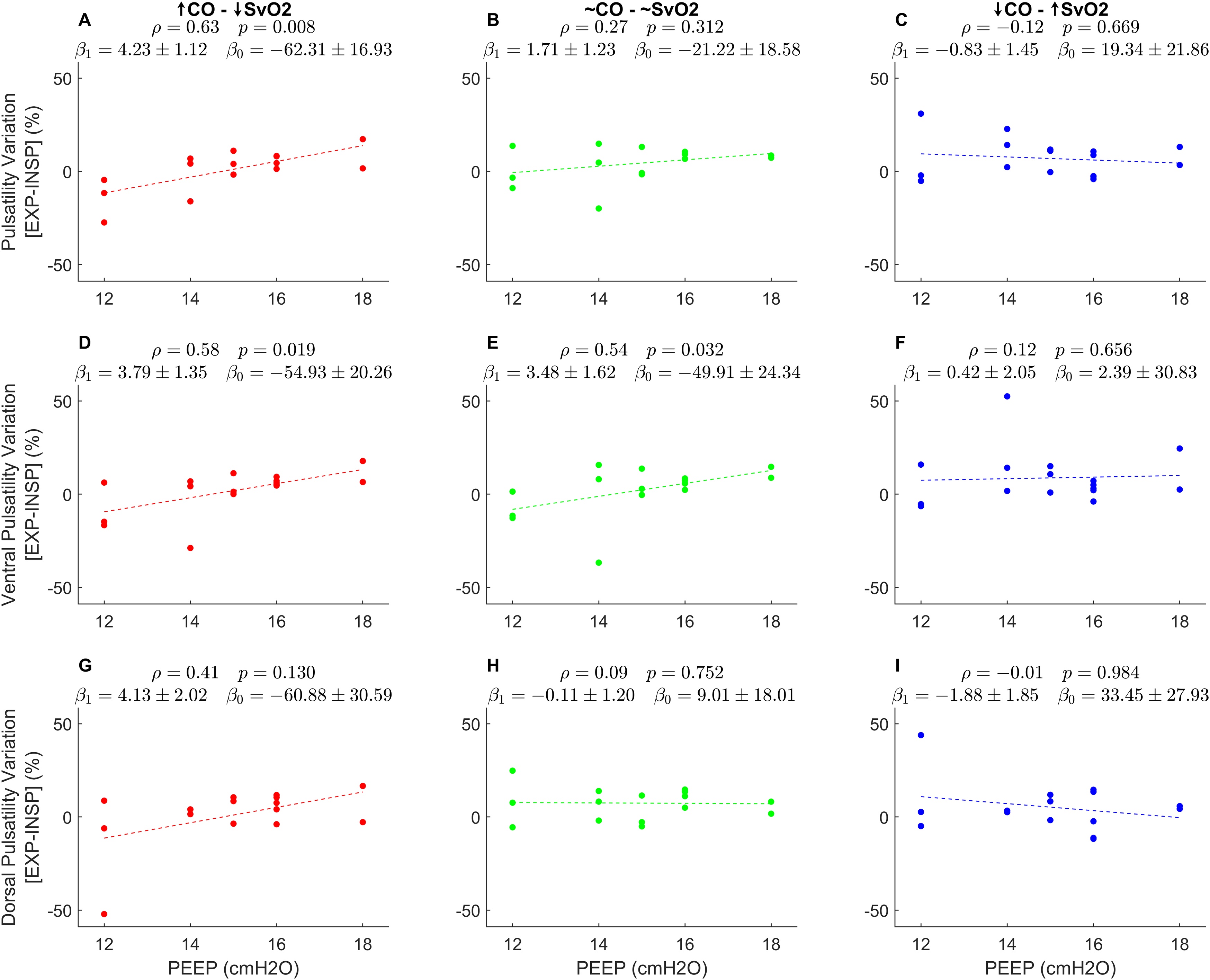
Relative changes in pulsatility. The difference between end-expiratory and end-inspiratory pulsatility is expressed as a percentage of pulsatility during the entire respiratory cycle (see **Methods**). **A -** a dependence on positive end-expiratory pressure (PEEP) was evident in data taken from the step with lower (SvO2), corresponding to higher cardiac output (CO). The same relationship was not apparent at the steps with intermediate and higher SvO2, corresponding to intermediate and lower cardiac output, respectively (**B-C**). When analysing the ventral region of interest, the same correlation was significant both at the low and intermediate SvO2 ranges (**D-E**), but not at high SvO2 values (**F**). Finally, in dorsal lung regions (**G-I**), no correlation reached statistical significance. β_1_ and β _0_ are the coefficients of a linear regression fit in the form Y ∼ β _1_*X + β _0_, ρ is Spearman's regression coefficient.

The magnitude of the inspiratory decrease in pulsatility amplitude in the ventral lung was correlated with PEEP at the low (ρ = 0.59, p = 0.019) and intermediate (ρ = 0.54, p = 0.032) SvO_2_ steps, but not at the high SvO_2_ step (ρ = 0.12, p = 0.656). No such correlation was observed in the dorsal lung (Fig. 3).

### Pulsatility amplitude and pulmonary artery pressures

**Fig. 4:**
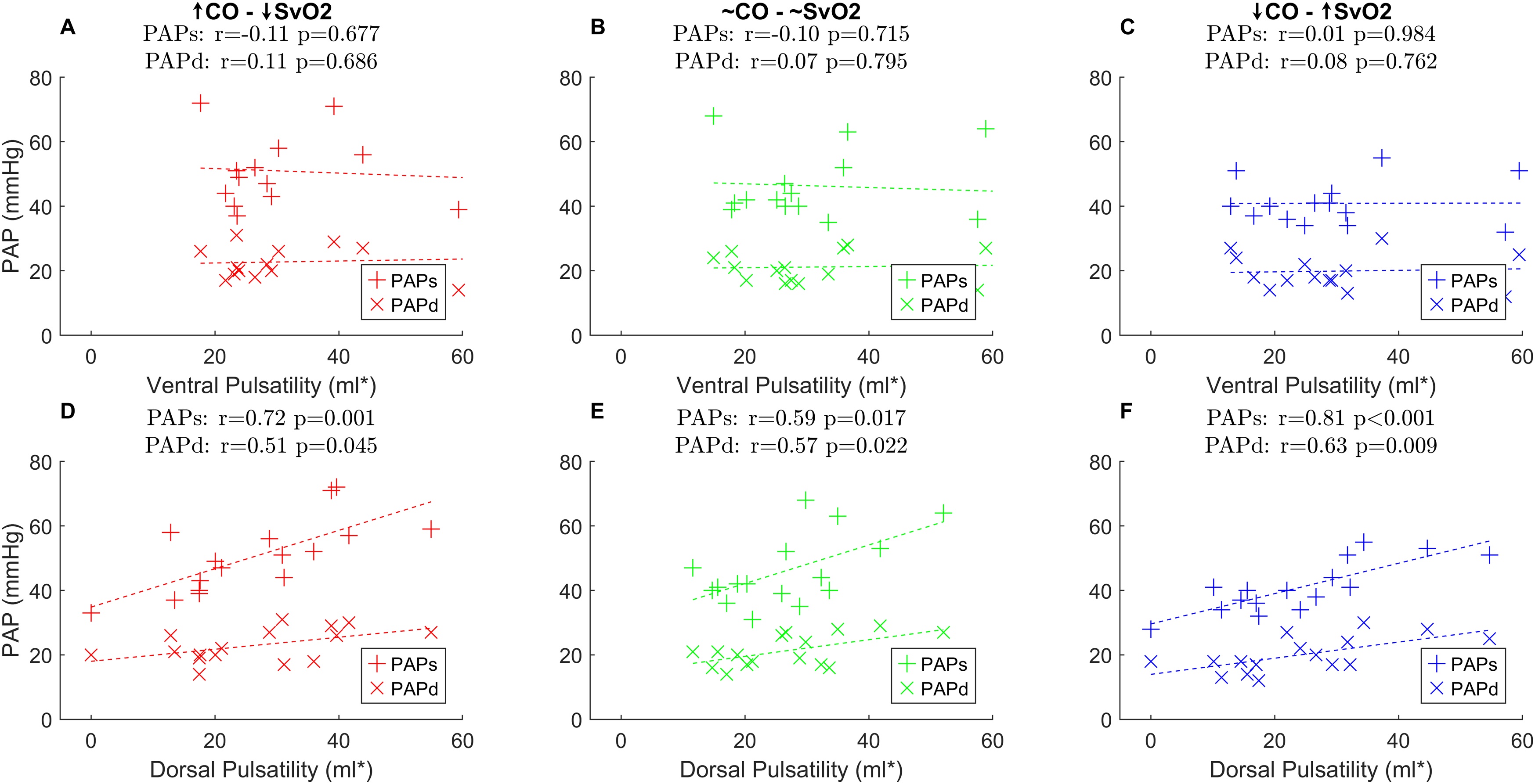
Correlation between pulsatility amplitude and pulmonary artery pressures (PAP). The *left column* corresponds to the lowest extracorporeal membrane oxygenation (ECMO) blood flow, targeting a mixed venous oxygen tension (SvO_2_) in the 70-75% range. The *middle column* refers to a 75-80% SvO_2_ target, while the *right column* to a >80% target. For a detailed description of the three steps, see text and captions for Fig. 2. **A-C –** no clear relationship was observed between ventral pulsatility amplitude expressed in ml of tidal volume (ml*) and diastolic (PAPd, “x” marker) or systolic (PAPs, “+” marker) pulmonary artery pressures (PAP). **D-F** – a direct correlation between pulsatility amplitude in the dorsal lung and was observed at all steps. r stands for Pearson's correlation coefficient.

In the dorsal lung, there was a correlation between pulsatility amplitude and both systolic (ρ = 0.72, p = 0.001 at low SvO_2_; ρ = 0.59, p = 0.017 at intermediate SvO_2_; ρ = 0.81, p < 0.001 at high SvO_2_) and diastolic (ρ = 0.51, p = 0.045 at low SvO_2_; ρ = 0.57, p = 0.022 at intermediate SvO_2_; ρ = 0.63, p = 0.009 at high SvO_2_) pulmonary artery pressure (Fig. 4D–F). The same correlations were not observed in the ventral lung (Fig. 4A–C).

### Pulsatility changes across SvO2 steps

**Fig. 5:**
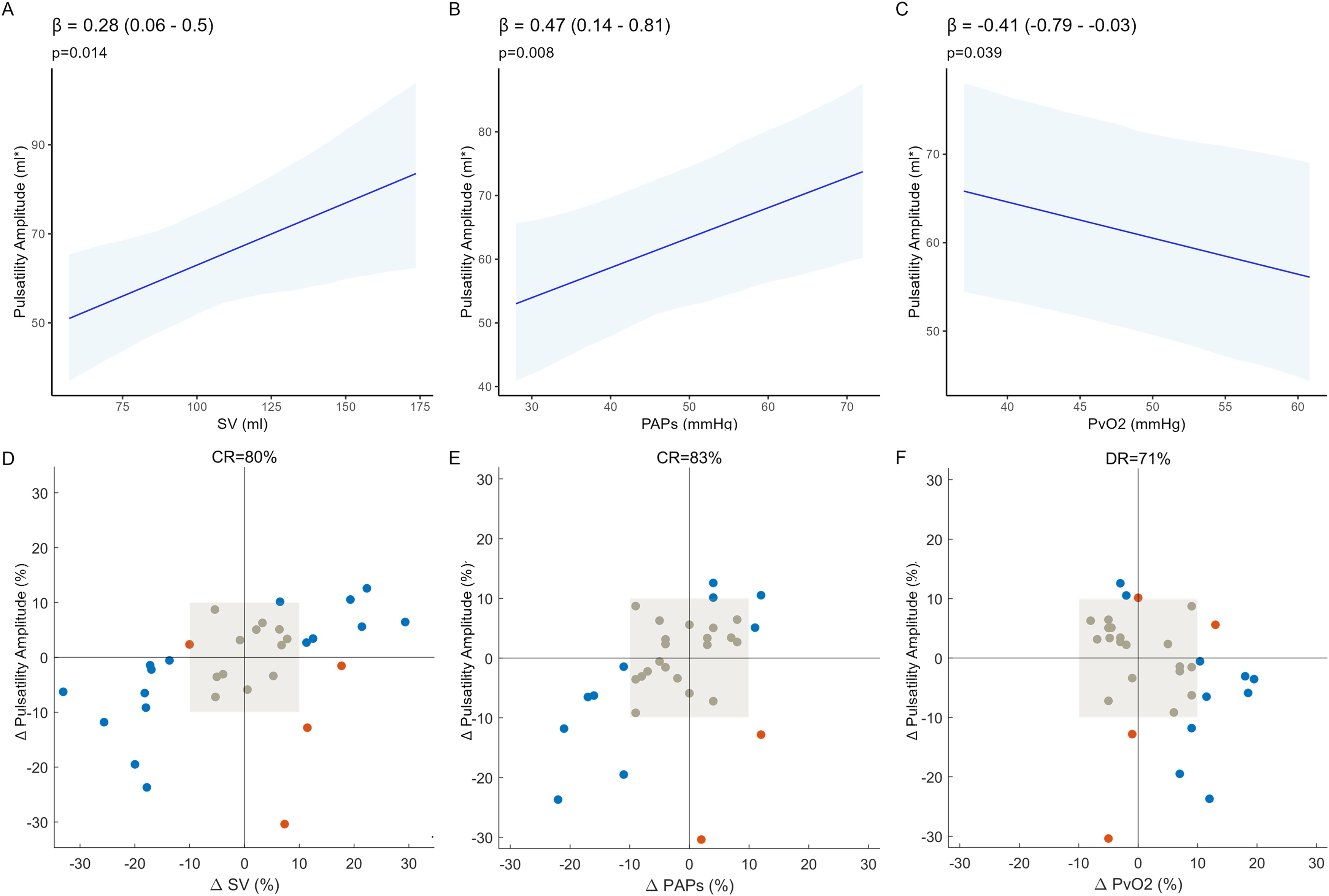
Effect of changing ECMO blood flow on pulsatility. Mixed-effect models were fitted to our data. Fixed-effect slopes and their confidence interval are shown in panels **A-C**. A direct relationship between pulsatility and stroke volume (**A**) and systolic pulmonary artery pressure (PAPs) (**B**), and an inverse relationship with PvO₂(PvO_2_) (**C**) were observed. **D-F** - The respective concordance rate (CR) between the relative changes of these same quantities, given a 10% exclusion zone, is portrayed in the three 4-quadrant plots. For PvO_2_, a discordance rate (DR) was calculated in a way analogous to the concordance rate, but counting discordant changes in the two quantities. β stands for the fixed-effect coefficient of the mixed-effect model. p-values were obtained by F-tests by employing Satterthwaite's approximation [39].

Mixed-effects modelling demonstrated that pulsatility increased with stroke volume (β = 0.28 (0.06−0.50) ml*/mL; p = 0.014) and pulmonary artery pressures (PAPs: β = 0.47 (0.14−0.81) ml*/mmHg; p = 0.008; PAPd: β = 1.29 (0.30–2.28) ml*/mmHg; p = 0.012) and decreased with mixed venous oxygen tension (PvO_2_: β = −0.41(−0.79 to −0.03) ml*/mmHg; p = 0.039) across the three SvO_2_ steps (Fig. 5A–C, Figure S1). The concordance rate was 80% between relative changes in pulsatility ampitude and stroke volume and 83% between pulsatility amplitude and pulmonary artery pressure, while changes between pulsatility amplitude and mixed venous oxygen tension (PvO_2_) were discordant in 71% of cases (Fig. 5D–F). However, the stroke volume effect only accounted for 5.7% of total variability in pulsatility amplitude and pulmonary artery pressures for 4.7% (systolic) and 8.3% (diastolic). PvO_2_ values accounted for 0.9% of total variability.

## Discussion

We observed that pulsatility changes were directly associated with stroke volume and pulmonary artery pressure and inversely with PvO_2_, with fair concordance between relative changes. In between-patient analyses, stroke volume contributed less to pulsatility amplitude, while a moderate-to-strong correlation between dorsal lung pulsatility amplitude and pulmonary artery pressure was evident. A consistent inspiratory decrease in pulsatility was also observed. These findings indicate that EIT-derived pulsatility reflects a complex interaction of physiological mechanisms and may serve as a non-invasive tool to assess pulmonary vascular alterations and RV afterload in patients with ARDS.

Vessel pulsation stems from a transient mismatch between blood flow into the arterial system and out towards more peripheral vascular segments. This framework has been widely adopted in models of the pulmonary circulation [37,45,46]. Consequently, any mechanism increasing downstream pressure or resistance is expected to increase vascular pulsatility and decrease outflow. However, it is also true that any increase in inflow or in vessel compliance [13] is also expected to increase vascular pulsatility.

We reasoned that acute changes in arterial compliance are unlikely in ARDS patients [19,47]. Instead, we hypothesized that downstream resistance/obstruction to outflow — be it due to a Starling or classical resistor — is the mechanism explaining the observed rise in both pulsatility and pulmonary arterial pressure [15,22,23].

Several factors influence pulmonary flow and can increase pulmonary artery pressure and RV afterload in ARDS patients. Most notably, West non-Zone 3 conditions [15], thrombosis [16], hypoxic vasoconstriction [17] and, possibly, increased interstitial pressure [48,49]. West non-Zone 3 conditions occur due to the proximity of some pulmonary vessels to the alveoli, that act as a Starling resistor [14], whereas hypoxic vasoconstriction and vasoconstriction due to hypercapnia are mediated by smooth muscle [17]. Intravascular thrombosis reduces total cross-sectional pulmonary vessel area. Although resulting from very different mechanisms, these phenomena all create a downstream flow "obstruction" relative to the more proximal, compliant segments of the pulmonary circulation, which are likely responsible for vessel pulsation [13].

This framework is consistent with previous observations in swine. Inducing unilateral lung atelectasis decreased regional blood flow, as measured by nuclear imaging and indicator-based EIT, but increased regional pulsatility in most animals of a previous experimental study [4]. More recently, a transient pulsatility increase shortly after injecting microspheres into the pulmonary circulation was observed [5].

Near complete obstruction of pulmonary vessels may cause retrograde flow from atelectatic to healthy lung regions, a previously observed phenomenon [50], which some authors termed "pendelblut" [4]. While retrograde flow may be present, in more general terms our data in humans support the view that vessel obstruction may result in transient accumulation of blood within lung vessels, which in turn can be detected as an increase in EIT pulsatility, especially in the dorsal lung, which is expected to be extensively damaged and collapsed in severe ARDS [51]. This would explain the relatively weak relationship between pulsatility and stroke volume in our cohort and the relatively stronger relationship with pulmonary artery pressure.

To explain the decrease in pulsatility amplitude during inspiration, mainly in the ventral lung regions, it should be noted that, as previously modelled, pulsatility also depends on vessel inflow. While downstream pressure increases during inspiration in those lung units, stroke volume flowing into the pulmonary circulation decreases, due to the increase in RV afterload caused by the rise in transpulmonary pressure [15] and decrease in venous return due to the rise in pleural pressure [52]. Available evidence shows a decrease in pulmonary artery flow with an increase in PAP when airway pressure is raised [15,53]. We reason that the effect of decreased vascular inflow predominated in our analysis, leading to the observed inspiratory decrease in pulsatility amplitude. We also noted that the percent change in pulsatility during inspiration was correlated with PEEP when cardiac output (and thus venous return) was high. This relationship was attenuated at lower cardiac output. The variation in stroke volume caused by pleural and transpulmonary pressure swings may be amplified when the cardiac function curve is steeper, at low SvO_2_ values, and attenuated when the cardiac function is shifted downwards [54].

While the observed respirophasic variation in pulsatility amplitude may not be a direct reflection of West Zones 1 and 2, it is likely related [55]. Given the harms associated with ventilated, non-perfused lung regions [56,57], this would address a relevant clinical problem. It is also intriguing to note that changes in ECMO flow appeared to affect these respirophasic variations. If confirmed, this finding further suggests that optimization of ECMO settings, potentially guided by EIT, could be leveraged to modulate global and regional hemodynamics and enhance lung and right ventricular protection (see Figure S2 for a proof of concept).

Varying ECMO blood flow produced concordant changes in PAP, stroke volume [26] and pulsatility. While the observed changes are smaller than the inter-patient variability at baseline (see Figure S1), their concordance rate above 80% is remarkable and consistent with previous results in healthy volunteers [10] though slightly worse than previously observed in in pigs [41] and critically ill patients [11].

Our analyses suggest that a shift in paradigm be adopted when assessing EIT pulsatility in ARDS patients. While the potential of pulsatility amplitude is currently under investigation as a non-invasive tool for stroke volume estimation and trending [10,11,41,58], the lack of correlation between stroke volume and pulsatility may reflect expected pathophysiologic changes to the pulmonary circulation in ARDS rather than a limitation. Given that the RV plays a crucial role in ensuring adequate cardiac output, increases in right ventricular afterload may contribute to hemodynamic instability and interfere with tissue perfusion in critically ill patients [19]. More specifically, acute cor pulmonale is associated with increased mortality in ARDS patients [22] and uncertainty remains as to the optimal RV protective ventilation strategy [18,23,59].

Our analyses have some limitations. First and foremost, although our small but intensively monitored cohort of severe ARDS patients receiving veno-venous ECMO, with SvO₂ targets defined by an experimental protocol is well suited to explore the relationship between pulsatility, pulmonary artery pressure, and mixed venous oxygenation, our findings are not generalizable to a broader ARDS population.

We also note that our within-patient analyses resulted in small marginal R^2^ values, suggesting that most of the variation in pulsatility measured after changing ECMO blood flow is due to unmeasured factors. This may be partly attributed to the fact that the studied range of SvO_2_ and PvO_2_ values was relatively narrow, limiting the extent of the observed hemodynamic changes and of hypoxic vasoconstriction. On the contrary, between-patient analyses allowed us to explore a broad range of physiological states, particularly, of pulmonary artery pressure values, supporting the hypothesis that downstream flow obstruction plays a relevant role in determining dorsal pulsatility amplitude in this population.

We restricted our observations to pulsatility. We have previously reported no significant impact of ECMO blood flow on only-ventilated or only-perfused units analyzed with hypertonic saline-enhanced EIT, possibly due to opposing effects of SvO_2_ and cardiac output [60]. Although a direct comparison with pulsatility might have been valuable, proper comparison would require an accurate decomposition of the indicator bolus. This proved challenging in this cohort.

Detailed hemodynamics were not collected during inspiration and expiration. In this, we were limited by the secondary nature of our reanalysis. Respiration-related changes in pulsatility were not assessed exactly at end-inspiration and end-expiration, but nearby beats were averaged. However, stricter criteria would have been feasible with longer recordings, as averaging a sufficient number of beats is required for meaningful pulsatility analyses [7].

Heart motion is a possible source of pulsatility, especially in the ventral lung [61]. We recognise that this might have partly confounded our results, as this phenomenon may be affected by intrathoracic pressures, inducing respirophasic variations in pulsatility. Lung vessel size may also be affected by intrathoracic pressures during respiration. However, whether this should be viewed as a confounder, or as part as West Zone physiology, has yet to be elucidated and ultimately depends on the compressed vascular segment.

Our proposed algorithm is not suitable for real-time monitoring and would ideally require an ECG signal, which was not part of the original protocol. Although we applied strict quality criteria to pulse detection, our rejection threshold is a trade-off between trace quality and sample size. Since pulse detection had to be performed before applying our algorithm for pulse separation, harmonics overlap and background noise resulted in significant pulse interpolation in few instances. However, traces were carefully reviewed, and subsequent processing did not appear to be significantly affected.

Finally, we acknowledge that the calibration of EIT arbitrary units to ml of tidal volume (ml*) is unconventional. However, other methods for comparing impedance amplitudes between patients are currently lacking.

## Conclusion

In severe ARDS patients on ECMO, pulsatility amplitude is influenced by stroke volume variations induced by respirophasic changes in pleural and transpulmonary pressure as well as by mixed venous oxygen content, whereas increased pulsatility in the dorsal lung reflects downstream flow obstruction and elevated pulmonary artery pressures. In this context, electrical impedance tomography may assist in monitoring the risk of developing West non-Zone 3 conditions and RV loading.

## CRediT authorship contribution statement

**Marco Leali:** Conceptualization, Methodology, Software, Formal Analysis, Writing – Original Draft, **Elena Spinelli:** Conceptualization, Investigation, Writing – Original Draft**, Marco Giani:** Investigation, Writing – Review & Editing**, Bertrand Pavlovsky:** Investigation, Writing – Review & Editing**, Michela Di Pierro:** Investigation, Writing – Review & Editing, **Stefania Crotti:** Investigation, Writing – Review & Editing, **Alfredo Lissoni:** Investigation, Writing – Review & Editing**, Giuseppe Foti:** Investigation, Writing – Review & Editing**, Giacomo Grasselli:** Investigation, Writing – Review & Editing**, Tommaso Mauri:** Conceptualization, Investigation, Writing – Original Draft, Supervision**, Douglas Slobod:** Conceptualization, Investigation, Methodology, Formal Analysis, Writing – Original Draft, Supervision.

## Consent for publication

Not applicable.

## Funding

The Department of Pathophysiology and Transplantation, University of Milan, is funded by the Italian Ministry of Education and Research (MUR): "Dipartimenti di Eccellenza" Program, 2023–2027. The Article Processing Charges were covered by the Research Institute of the McGill University Health Centre (RI-MUHC).

## Ethics approval and consent to participate

The primary study was conducted in the general ICUs of Maggiore Policlinico (Milan, Italy) and San Gerardo (Monza, Italy) hospitals. The study protocol was approved by the ethical committees of the two participating centers (reference numbers 968_2020 and 3896, respectively), and informed consent requirements were met according to local regulations.

## Availability of data and material

The datasets and images used and/or analysed during the current study are available from the corresponding author on reasonable request.

## Declaration of competing interest

TM received financial support from Drager, Fisher and Paykel, Hamilton and Mindray for speaking at symposia. All other authors: none.

## Figures and Tables

**Figure:**
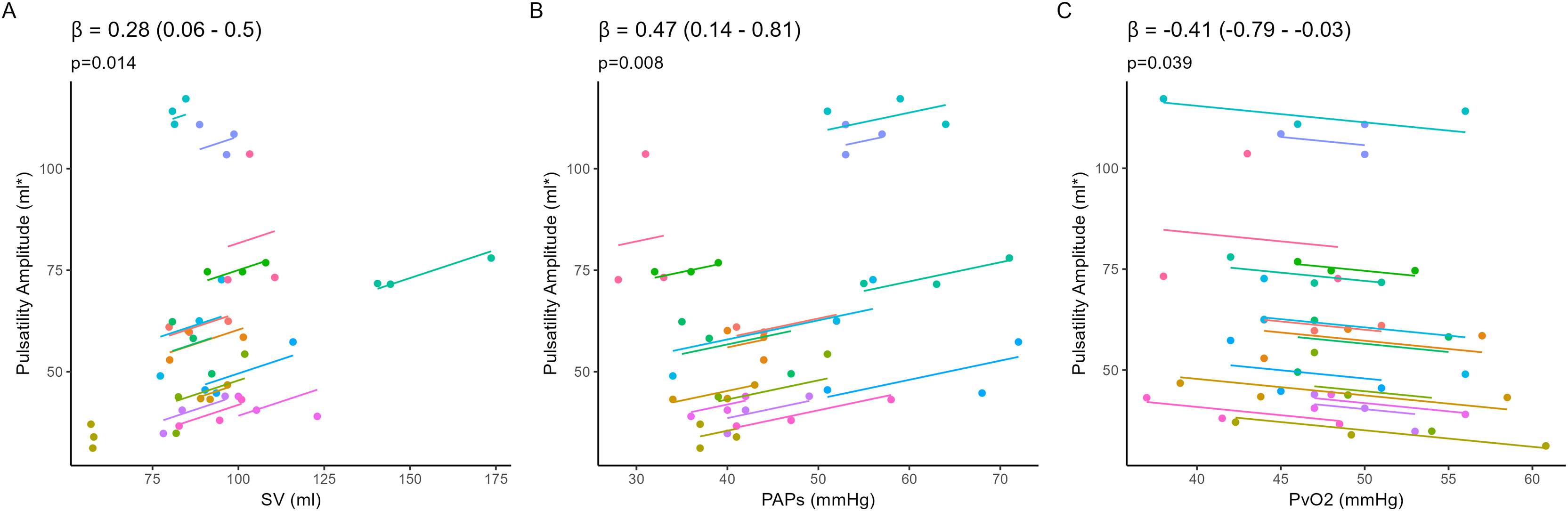


**Figure:**